# IHH enhancer variant within neighboring NHEJ1 intron causes microphthalmia anophthalmia and coloboma

**DOI:** 10.1038/s41525-023-00364-x

**Published:** 2023-08-14

**Authors:** Ohad Wormser, Yonatan Perez, Vadim Dolgin, Bahman Kamali, Jared A. Tangeman, Libe Gradstein, Yuval Yogev, Noam Hadar, Ofek Freund, Max Drabkin, Daniel Halperin, Inbar Irron, Erika Grajales-Esquivel, Katia Del Rio-Tsonis, Ramon Y. Birnbaum, Gidon Akler, Ohad S. Birk

**Affiliations:** 1 The Morris Kahn Laboratory of Human Genetics, National Institute for Biotechnology in the Negev and Faculty of Health Sciences, Ben-Gurion University of the Negev, Beer-Sheva, Israel; 2 Medical Advisory Committee, United Mashhadi Jewish Community of America, 54 Steamboat Rd., Great Neck, NY 11024 USA; 3 Department of Biology and Center for Visual Sciences, Miami University, Oxford, OH 45056 USA; 4 Department of Ophthalmology, Soroka Medical Center and Clalit Health Services, Faculty of Health Sciences, Ben-Gurion University of the Negev, Beer-Sheva, Israel; 5 Department of Life Sciences, Ben-Gurion University of the Negev, Beer-Sheva, Israel; 6 TOVANA Health, Houston, TX USA; 7 Precision Medicine Insights, P.C., Great Neck, NY USA; 8 Genetics Institute, Soroka Medical Center affiliated to Ben-Gurion University of the Negev, Beer-Sheva, Israel

**Keywords:** Hereditary eye disease, Gene regulation, Eye abnormalities, Genetic linkage study

## Abstract

Genomic sequences residing within introns of few genes have been shown to act as enhancers affecting expression of neighboring genes. We studied an autosomal recessive phenotypic continuum of microphthalmia, anophthalmia and ocular coloboma, with no apparent coding-region disease-causing mutation. Homozygosity mapping of several affected Jewish Iranian families, combined with whole genome sequence analysis, identified a 0.5 Mb disease-associated chromosome 2q35 locus (maximal LOD score 6.8) harboring an intronic founder variant in *NHEJ1*, not predicted to affect NHEJ1. The human *NHEJ1* intronic variant lies within a known specifically limb-development enhancer of a neighboring gene, Indian hedgehog (Ihh), known to be involved in eye development in mice and chickens. Through mouse and chicken molecular development studies, we demonstrated that this variant is within an *Ihh* enhancer that drives gene expression in the developing eye and that the identified variant affects this eye-specific enhancer activity. We thus delineate an *Ihh* enhancer active in mammalian eye development whose variant causes human microphthalmia, anophthalmia and ocular coloboma. The findings highlight disease causation by an intronic variant affecting the expression of a neighboring gene, delineating molecular pathways of eye development.

## Introduction

Most monogenic diseases are due to mutations in coding sequences^1^. However, with the emerging understanding of the functionality of non-coding sequences and the growing availability of whole genome sequencing, few cases of non-coding mutations have already been shown to cause monogenic phenotypes^2,3^. While such mutations are often within or adjacent to genes relevant to the disease phenotypes, the long-range effects of enhancers are such that genomic disease-causing variants might reside remotely from the affected gene; In fact, intronic, and even coding sequences of genes have been shown to act as enhancers of neighboring genes^4,5^.

Anophthalmia and microphthalmia are severe ocular malformations considered part of a phenotypic continuum with ocular coloboma, resulting from incomplete fusion of the choroid (or optic) fissure^6–8^. In severe bilateral anophthalmia or severe microphthalmia, the genetic cause is identifiable in ~80% of cases^9^. However, the genetic cause of other forms of microphthalmia, anophthalmia, and coloboma (MAC), particularly isolated coloboma, remains mostly unknown, due to our limited understanding of normal optic fissure molecular morphogenesis^9,10^. The estimated prevalence of anophthalmia, microphthalmia, and coloboma is 1 per 30,000, 1 per 7000, and 1 per 5000 live births, respectively, with higher incidence in specific consanguineous cohorts^9,11^. Iranian Jews comprise a small, ethnically distinct group founded some twenty-seven centuries ago^12,13^. Specifically, a cohort of Iranian Jews, originating from the vicinity of Mashhad in north Persia, have remained relatively isolated, with a very high degree of inbreeding^13,14^. A high incidence of putatively autosomal recessive isolated MAC was noted in this ethnic group almost three decades ago, with significant inter-and intra-patient phenotypic variability on this continuum^15^. Although those observations suggested a founder mutation, we and others have failed over the past two decades to find the pathogenic variant, using the tools available at the time^7^.

Through studies of several affected consanguineous Iranian Jewish kindreds, not directly related to the ones previously described^7,15^, we now identify a MAC disease-associated 0.5 Mb genomic locus on chromosome 2q35 (maximal LOD score 6.8) containing a single nucleotide variant that lies within an intron of *NHEJ1*. Through chicken and mouse experiments, we demonstrate that the intronic *NHEJ1* sequence acts in eye development as an enhancer of the neighboring *Ihh* gene, which is inactivated by the variant.

## Results

### Clinical studies and homozygosity mapping

**Fig. 1:**
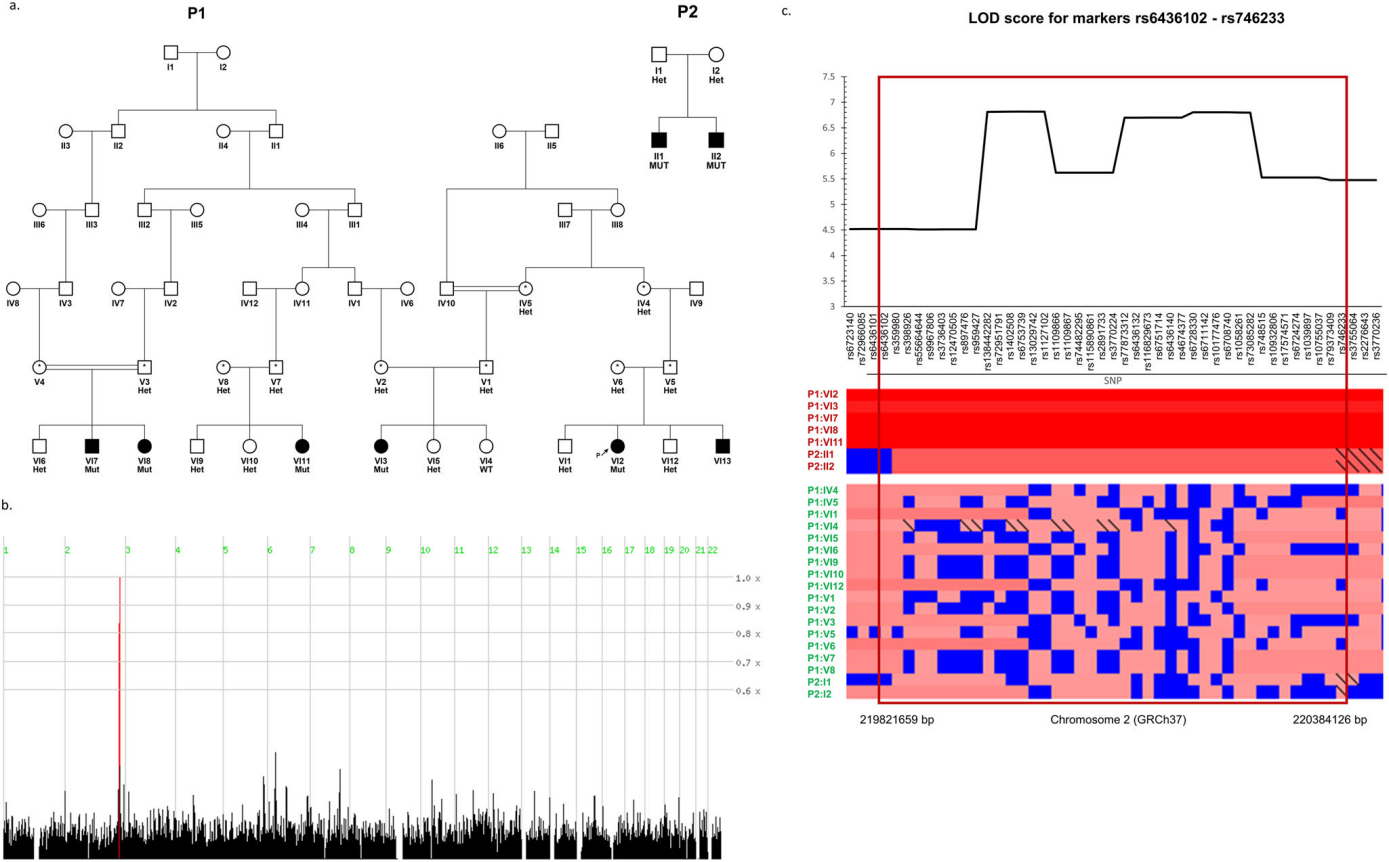
Pedigrees, linkage analysis, and segregation. **a** The two pedigrees of Iranian Jewish kindred studied (P1 – pedigree 1, P2 – pedigree 2). Affected individuals (blackened squares or circles) had a spectrum of congenital eye malformations, ranging from mild optic nerve coloboma to microphthalmia and anophthalmia. DNA samples were obtained from all individuals appearing with genotype of the *NHEJ1* (NM_024782.2): c.588+18131 A > G variant (‘Het’= heterozygous, ‘WT’ = homozygous wild type, ‘MUT’ = homozygous mutant, *= obligate carrier). **b** Genome-wide homozygosity mapping for both pedigrees together, using 302,692SNP arrays, was carried out using Homozygosity-Mapper. The only region where all and only the affected individuals have runs of homozygosity is labeled in red. **c** In the red rectangle- the genotypes plot for the part of the region marked in red in (**b**), spanning chr2:219,821,659-220,384,126 (GRCh37) and containing markers rs6436102 to rs746233. Upper plot: the logarithm of odds (LOD) across the locus (multipoint analysis, both analyzed pedigrees combined, calculated using SUPERLINK online and depicted using Microsoft-Excel). The maximal LOD score for this locus at chromosome 2 was 6.8169 at rs6753739. The lower plot (below the markers/SNPs that head the columns) presents all studied individuals’ genotypes at this locus and marks the shared ancient homozygous haplotype borders. Orientation for the genotypes plot (Homozygosity-Mapper genotypes view): the affected individuals are displayed on the upper lines and the unaffected at the bottom lines, with a small free space between them. A blue box represents heterozygosity for this SNP (‘Aa’), and different shades of red reflect the length of the stretch of homozygous SNPs (‘AA’) for homozygous genotypes. Markers homozygous for the minor allele (‘aa’ based on this study) have a black diagonal line.

We studied two consanguineous pedigrees of Jewish Iranian ancestry originating from the Mashhad region (Fig. 1a), with multiple offspring suffering from a spectrum of isolated ocular manifestations, ranging from a milder phenotype of optic nerve coloboma to a severe phenotype of microphthalmia and even anophthalmia. Most affected individuals had a phenotype in the severe part of the spectrum, although the ocular features varied among patients and within the same patient (Table 1). For example, in pedigree 2, patient II-1 had anophthalmia of the right eye and optic nerve coloboma in his left eye. None of the affected individuals had skeletal or cranial abnormalities.

**Table 1:** Affected patients’ phenotypes.

Pedigree	Individual	Age at last assessment (years)	Phenotype
P1	VI-7	30	Microphthalmia OS>OD. Optic nerve coloboma OU. High myopia with myopic chorioretinal degeneration and retinal tears OU. Nuclear cataract OU. Strabismus OS, nystagmus OU.
P1	VI-8	20	Anophthalmia OS. Microphthalmia and retinal detachment OD with complete vision loss by age 8y. At 20y, has bilateral ocular prostheses.
P1	VI-3	18	Anophthalmia OS. Severe microphthalmia, very small and scarred cornea and glaucoma OD. No residual retinal responses on electroretinography. At 18y completely blind with ocular prostheses OU.
P1	VI-2	3.9	Microphthalmia and microcornea OS. Colobomata of the optic nerve and adjacent chorioretina OS>OD. Retinal pigmentary alterations OU. Absent macular architecture OS. High myopia and atrophic retinal pathches OD. Strabismus (esotropia) OS. Nystagmus OU.
P1	VI-13	0.9	Microphthalmia OD and coloboma OU.
P1	VI-11	9	Microphthalmia and developmental delay.
P2	II-1	3	Anophthalmia and ocular prosthesis OD. Optic nerve coloboma, myopia and nystagmus OS.
P2	II-2	1.4	Optic nerve coloboma OU. Very high myopia OS>OD. Retinal atrophy OU. Nystagmus OU.

*OD* right eye, *OS* left eye, *OU* both eyes, *y* years.

Chromosomal microarrays (CMA), done for several affected individuals of both pedigrees, were normal. Whole exome sequencing identified no coding sequence variants or predicted splice site mutations that could explain the phenotype (data not shown). Genome-wide homozygosity mapping identified a ~1.5 million base pairs (Mbp) homozygosity locus on chromosome 2q35 in pedigree 1 (Sup. Fig. 1). Several homozygosity loci were found in pedigree 2, including a ~1.1 Mbp locus on chromosome 2q35 (Sup. Fig. 1). Using genotype data from the 25 available family members of both pedigrees analyzed together, we identified a minimal 0.5 Mbp shared haplotype (Fig. 1 and Sup. Fig. 1): all seven available affected individuals of both pedigrees had shared homozygosity on chromosome 2q35 between markers rs6436102 and rs746233, corresponding to chr2:219,821,659-220,384,126 (GRCh37; Fig. 1 and Sup. Fig. 1). Maximal LOD score was 6.8169 at rs6753739 (multipoint, combined analysis of both pedigrees; Fig. 1c and Sup. Fig. 1).

### Identification of the NHEJ1 intronic variant

**Fig. 2:**
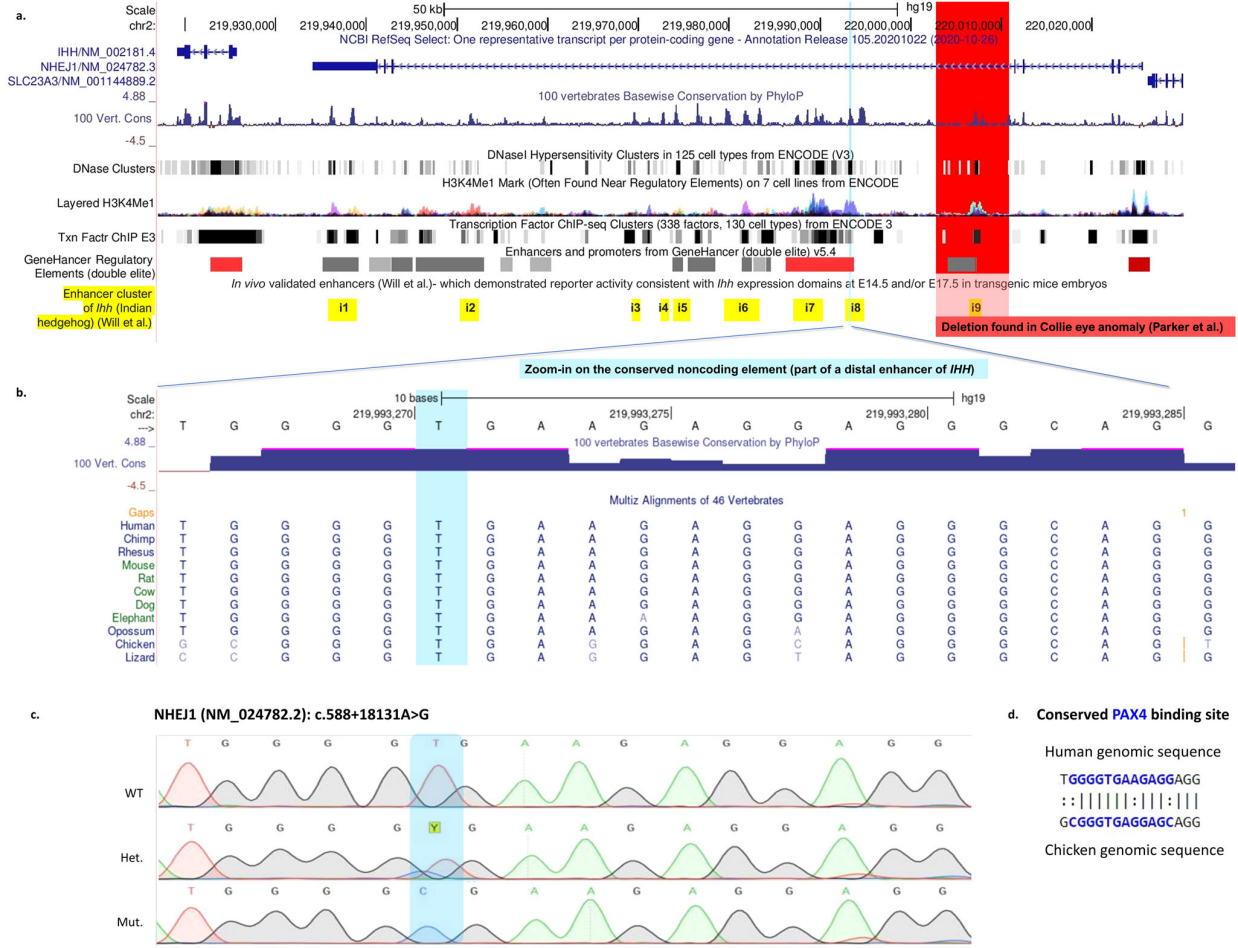
Human and dog coloboma-linked mutations found within validated *Ihh* enhancers. **a** The genomic landscape of *NHEJ1* and *IHH* from the UCSC Genome Browser. The tracks presented are publicly available ones associated with *cis-*regulatory elements: histone marks (H3K4me1), the open chromatin state (DNaseI hypersensitivity), and the evolutionary conservation, as well as other data from GeneHancer and ENCODE, are given in detail in Sup. Fig. 3. In yellow, the nine *Ihh* enhancers tested in mice by Will et al.; In red- the approximate location of the 7.8 kb deletion found in dogs (by Parker et al.) as the likely causal variant for Collie Eye Anomaly (corresponding to “macular colobomas” in humans). UCSC’s BLAT was used to locate the enhancers and the 7.8 kb deletion in dogs on the human genome (hg19). In light blue- the variant found in the present study. **b** Zoom-in on the proximal end of one of *Ihh*’s distal enhancers- ‘i8’ (name termed by Will et al.), presented with evolutionary conservation track. In light blue- the variant found in the present study. **c** Sanger sequencing demonstrating the single nucleotide substitution- *NHEJ1* (NM_024782.2): c.588+18131 A > G, found in all available affected individuals in our study. WT unaffected homozygous wild-type individual; Het. obligatory heterozygous carrier; Mut. affected homozygous mutant individual. **d** Conserved PAX4 binding site (in blue), found using rVista, in the Human and the Chicken genomic sequences.

As whole exome sequencing identified no coding sequence variants in genes previously associated with MAC, or in any coding sequences within the chromosome 2 locus, Whole Genome Sequencing (WGS) was performed for an affected patient (P1:VI-2) and his parents (P1:V-5&V-6). The average sequencing depths for the P1:VI-2, P1:V-6, and P1:V-5 samples were 42.84X, 38.38X, and 31.95X, and the sequence coverage of the target genome was 99.14%, 99.86%, and 91.14%, respectively. The WGS trio data within the 0.5 Mbp locus were analyzed using Ingenuity Variant Analysis software (QIAGEN, Redwood City, CA, USA), and Integrative Genomics Viewer (IGV). All the single nucleotide variants, indels, and micro-deletions in the chromosome 2 locus, which were found to be rare (<1% ExAC, gnomAD, NHLBI-ESP, and 1000 Genomes) and in homozygous state only in the proband- P1:VI2 (but not in his parents or in-house WGS controls), were eligible for analysis. Of 13 identified variants, six were found to be frequent (more than 1.379%) in a subpopulation of public databases (specifically, gnomAD Jewish frequency; Sup. Table 1). Only one variant of the remaining seven was found to be evolutionary conserved, with a striking phyloP *p*-value of 0.000001403 (the others were not conserved and had no phyloP p-value at all) (Sup. Table 1 and Sup. Fig. 2). This variant, *NHEJ1* (NM_024782.2): c.588+18131 A > G (NG_007880.1: g.37317 A > G), submitted to ClinVar (SCV002769719), has no reported frequency in gnomAD. Sanger sequencing and RFLP analysis verified that the *NHEJ1* intronic variant segregated according to the phenotype within both pedigrees as expected for recessive heredity (Figs. 1a, 2). RFLP screening of 87 ethnically matched control samples identified no other individuals heterozygous or homozygous for this variant (data not shown).

### The NHEJ1 intronic variant disrupts an eye-enhancer of the neighboring IHH gene

Based on high evolutionary sequence conservation, the intronic *NHEJ1* variant was predicted to be ‘possibly deleterious’ by some algorithms (CADD score of 18.68; ‘disease causing’ with a probability of 100% by MutationTaster), despite the miniscule chance that it can affect any protein features of NHEJ1. Splicing prediction algorithms (such as Human Splicing Finder) predicted no significant splicing signal impact. As the intronic variant was not predicted to impact *NHEJ1* coding sequence or expression, we set out to identify possible effects of the variant on regulatory sequences of neighboring genes. Possible roles as a regulatory element were supported by MutationTaster regulatory features analysis (attributed to histone 3 Lysine residues 9, 4, and 36 methylations and DNase1 hypersensitive site). Furthermore, OMIM review of the gene (611290), together with GeneHancer (element GH02J219121) and ENCODE (element EH38E2075899), pointed to possible regulatory effect on the neighboring gene, *IHH*.

UCSC’s Genome Browser was used to visualize the relevant intron in *NHEJ1*, along with publicly available *cis-*regulatory elements associated tracks: the enhancer histone mark H3K4me1, the open chromatin state (DNAseI hypersensitivity), and evolutionary conservation (Fig. 2). It became evident that the intronic sequence contains several regions of high evolutionary conservation which are accessible for regulatory factors, mostly on blood and liver-derived cell lines (Fig. 2 and Sup. Fig. 3). These conserved non-coding elements (CNE) were previously described by Will et al.^16^, who found that most of them were part of the enhancer cluster of *Indian hedgehog (Ihh)*, affecting *Ihh* mRNA levels in several mouse tissues. Those previous studies focused on limb development and did not test possible effects of these enhancers on eye development. We showed that the variant we identified in *NHEJ1* was within a CNE that matches one of the identified enhancers, ‘i8’ of *Ihh*^16^ (Fig. 2 and Sup. Fig. 3). Using ChIP-seq data from ENCODE, a number of transcription factors were found to cluster around the variant location, mostly in K562 cells (acute erythroid leukemia cell line). In addition, using rVista^17^, one predicted transcription factor binding motif, for PAX4, was identified at the exact variant location (Fig. 2d).

We hypothesized that the MAC-causing intronic variant alters Ihh enhancer activity affecting eye development, as extracellular signaling through Ihh is known to take part in eye development in mice^18,19^. *Ihh* is expressed outside the mouse developing eye between embryonic days 11 and 14, adjacent to the retinal pigment epithelium (RPE) component of the developing choroid, and is directly required for Hedgehog (Hh) target gene expression in the periocular mesenchyme (POM) and for normal pigmentation pattern of the RPE^18,20^. The retina–POM interaction is fundamental to fissure closure^21^, and haploinsufficiency of *Ihh* leads to the “Creeper” phenotype in chicken, with microphthalmic and colobomatous eyes, along with phocomelic limbs^21,22^ (Sup. Fig. 2).

Interestingly, prior studies on the cause of canine macular coloboma, an autosomal recessive disease named Collie eye anomaly (CEA), identified a nearby disease-associated 7.8k base pairs deletion (chr37:28697542-28705340 according to the Dog genome established, May 2005, Broad/canFam2) within the same intron of the canine ortholog of *NHEJ1*^23^ (Fig. 2). Using UCSC’s Genome Browser, we located this 7.8kbps intronic deletion as the neighboring CNE (chr2:220002923-220010954 according to hg19/GRCh37, 86.7% identity) and noted that it encompasses another experimentally verified enhancer of *Ihh (*in mice), termed ‘i9’ by Will et al.^16^. This group found that the deletion of enhancers ‘i7’-‘i8’-‘i9’ resulted in 60% reduction of *Ihh* mRNA levels in the tissues they studied: forelimb, growth plate and skull. However, contrary to other deletions of enhancers they described, mice homozygous for this deletion continued to have *Ihh* expressed in their fingertips, and only a slight effect on the skeleton was noticed^16^. These data suggested that both the ‘i8’ and i9 *Ihh* enhancers might affect *Ihh* levels; the human and canine variants suggested that these enhancers might be relevant to tissues other than skeletal, such as the developing eyes, not analyzed by Will et al.

**Fig. 3:**
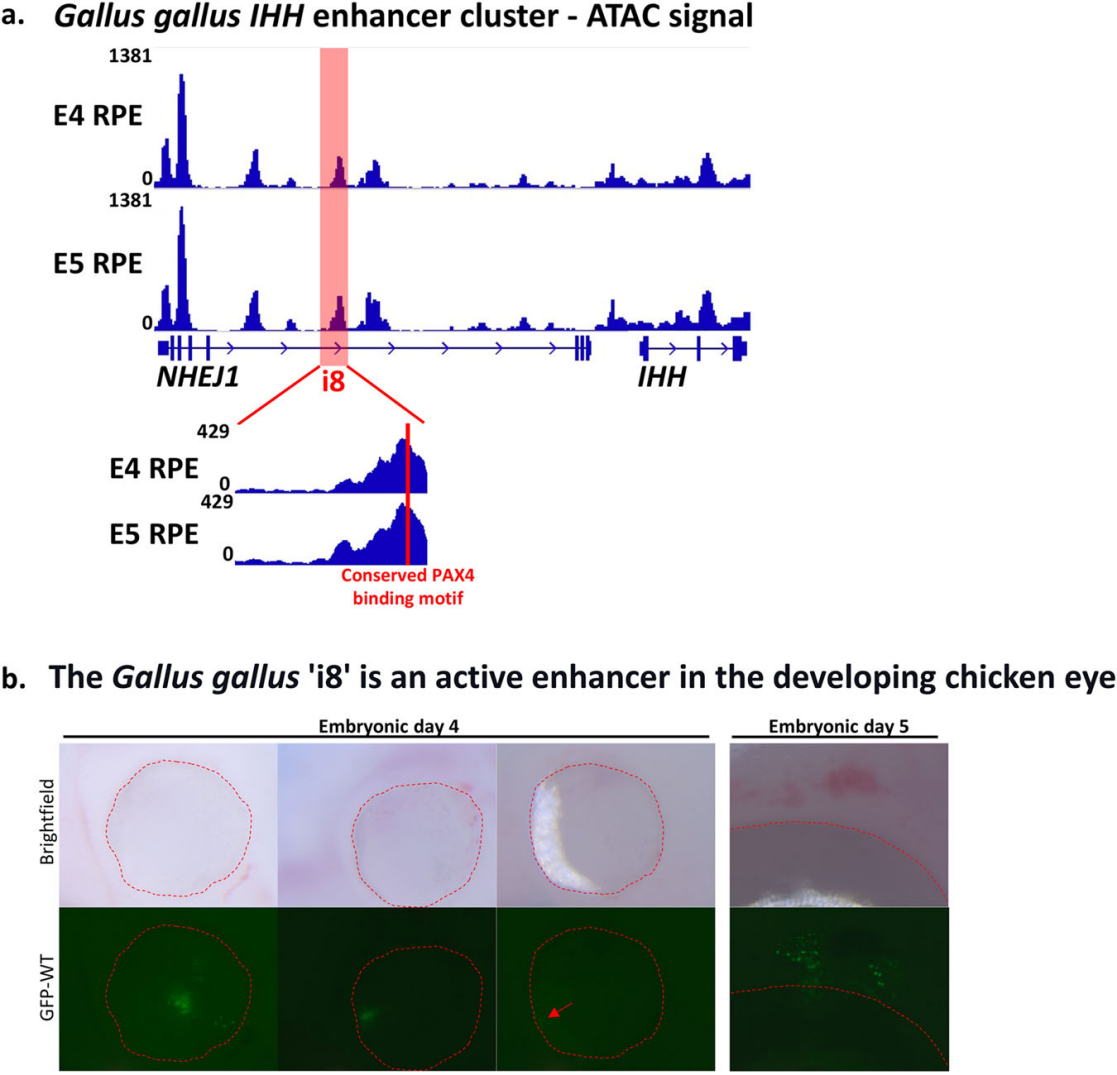
Ihh enhancer is conserved and active in the native context of the Chicken developing eye, and ‘i8’ is sufficient to drive GFP expression in the developing Chicken eye. **a** ATAC-seq was performed using RPE cells from embryonic day 4 (E4) and embryonic day 5 (E5) chicken embryos. Genome browser tracks display the accessibility signal across the *NHEJ1* and *IHH* loci. The region highlighted in red corresponds to the conserved i8 enhancer, which overlaps with a prominent peak that is characteristic of an active genomic regulatory element. Below, a panel displays an enlarged view of the i8 region, and the location of the predicted PAX4 binding motif is indicated by a vertical red bar. **b** The *Gallus gallus* enhancer construct (gal-i8-ptkEGFP ver2) was injected into the optic vesicles of Hamburger-Hamilton stage 9–12 chicken embryos post-incubation) with an ECM 830 High Throughput Electroporation system. Punctate fluorescence was observed 2- and 3-days post-electroporation (Embryonic day 4 and 5 respectively) and visualized. The periphery of the eye is outlined with a dashed line. Red arrow points to a region of dim GFP fluorescence. A total of n = 5 embryos (4 with gal-i8-ptkEGFP ver2) displayed enhancer activity throughout the study.

In order to further investigate the activity of the *Ihh* enhancer cluster during ocular development, we performed ATAC-sequencing on RPE cells within the developing chicken (Fig. 3a). For this assay, we analyzed RPE at embryonic days 4 and 5 of development (Hamburger Hamilton stages 24 and 26), which represents a developmental window during which the optic fissure has not yet closed and the POM and RPE are differentiating^24^, and thus the putative *IHH* enhancer cluster could be active. Interestingly, we observed chromatin accessibility broadly across the *NHEJ1* and *IHH* loci, including peaks localized to the promoter and intronic regions of these genes. Notably, a sharp accessibility peak coincided with the conserved i8 enhancer region, suggesting that this locus represents a regulatory element that is active during this developmental timeframe. Moreover, the summit of the observed i8 peak overlaps with the conserved PAX4 binding motif and the orthologous site of the c.588+18131A > G variant, which reinforces the premise that this particular nucleotide sequence is involved in the recruitment of regulatory proteins, such as transcription factors. Thus, we contended that the i8 enhancer region represents a conserved and active enhancer region in the developing chicken RPE and has the potential to regulate gene expression during early eye morphogenesis. Further studies are in place for delineation of the specific factors whose binding is affected by the variant.

Since the human intronic variant we identified and the CEA canine attributed deletion are both located within or encompass putative *Ihh* enhancers, we went on to study possible activity of the enhancers in eye development. First, we studied transgenic chicken embryos to test whether the ‘i8’ enhancer acts in determining eye-specific expression. The optic vesicles and anterior neural tube of embryonic day 2 chicken embryos (Hamburger-Hamilton stage 9–12) were injected and electroporated with the chicken orthologous ‘i8’ enhancer (gal-i8-ptkEGFP ver2 or gal-i8-ptkmCherry ver2). Fluorescence was observed at embryonic day 4 or 5. Punctate fluorescence was observed in the POM (adjacent to the forming RPE) and across the surface of the eye in a number of the embryos (*n* = 5), demonstrating that ‘i8’ is active as an enhancer in the developing eye of the chicken (Fig. 3b; Sup. Fig. 4a). No embryos electroporated with a control plasmid lacking the enhancer sequence (ptk-EGFP) exhibited fluorescence (*n* = 19, Sup. Fig. 4b).

**Fig. 4:**
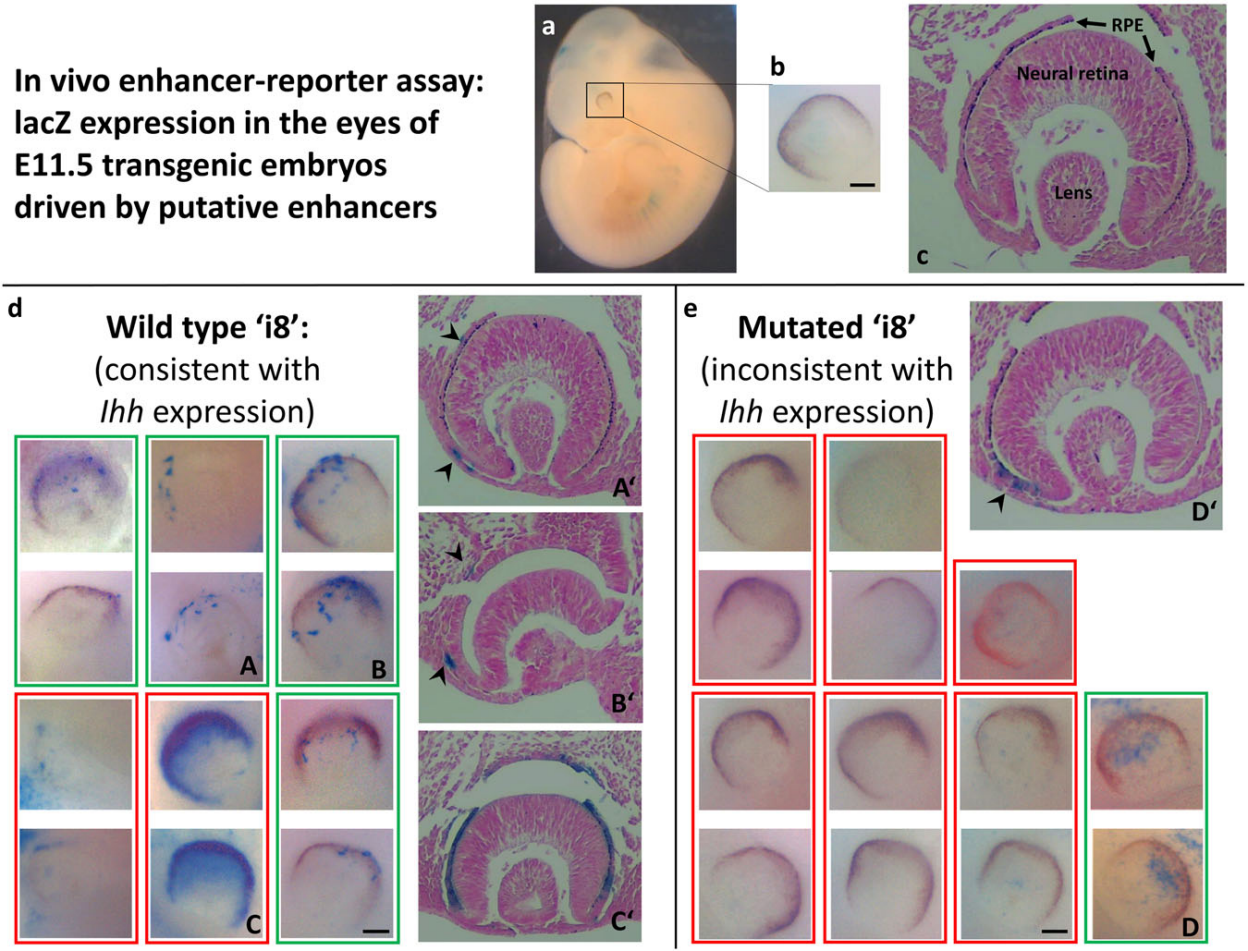
Only the WT ‘i8’ *Ihh* enhancer activity phenocopies *Ihh* eye expression in mouse embryos. In vivo enhancer-reporter assay was applied to compare with the known expression pattern of *Ihh* in the developing eyes of embryonic day E11-12 mouse embryos (Dakubo et al.^18^). Three putative *Ihh* enhancers were cloned and inserted into Hsp68 minimal promoter - lacZ reporter vectors: two previously identified limb *Ihh’s* enhancers of the mouse genome (termed ‘i8’ and ‘i9’ by Will et al.^16^), and a mutated version of ‘i8’ (site-directed mutagenized to contain the exact single-nucleotide variant found in the patients). A negative control vector, without a putative enhancer, was also tested. **a**, **b** Whole mount embryo and zoom-in of one of its eyes. **c** Normal E11.5 developing eye, coronal section (nuclear fast-red counterstain; no detectable X-Gal stain). **d**, **e** Panels showing all eyes of the wildtype (WT) ‘i8’ (**d**) and the mutated ‘i8’ transgenic embryos (**e**); both in (**d**) and in (**e**), upper and lower images in each red/green rectangle represent both eyes of the same samples, when available. An additional sample with non-anatomically localized staining, thus considered to be an artifact, is shown in Sup. Fig. 5c. Positive X-gal staining, which phenocopies *Ihh* expression at the POM (green rectangles) and is consistent in more than three of at least five embryos in the developing eyes of the transgenic embryos, was noted only for the WT ‘i8’ enhancer Subfigures A’,B’,C’,D’ depict histology sections of A,B,C,D. Note- several samples, mainly the two samples in red rectangles in panel D, were considered negative as it doesn’t phenocopy *Ihh* expression, although they may present under/overexpression, possibly due to positional effects. The scale bar represents 0.1 mm.

Based on these findings, we went on to study a model system closer to human eye development, looking at the effects of the wild-type and mutant enhancer sequences on eye development in the mouse. We applied the same in vivo enhancer-reporter assay previously described^4,25^ to test the ability to drive gene expression in the developing eye by ‘i8’ and ‘i9’, as well as a mutated ‘i8’ enhancer harboring the human variant in the mouse enhancer ortholog. To that end, we repeated the studies of Will et al., and cloned two of the previously identified *Ihh* enhancers (termed ‘i8’ and ‘i9’) from the mouse genome into a heat shock protein (Hsp) 68 minimal promoter - lacZ reporter vector^16^. We added a mutated version of ‘i8’ (point-mutated to contain the exact variant found in our patients) and sequence-verified all constructs. An empty vector without a putative enhancer was used as a negative control (Sup. Table 2). We expected the reporter gene, activated by the *Ihh* enhancers, to phenocopy the expression pattern of the endogenous *Ihh*, and more specifically- to appear in patches of non-pigmented cells adjacent to the RPE outside the developing eyes of E11.5 mouse embryos^18^. The vectors were linearized, underwent pro-nuclear injections to fertilized mouse oocytes and implanted in pseudo-pregnant female mice. Embryos were collected on E11.5, genotyped and stained for LacZ. At least 5 PCR and lacZ -positive embryos were generated for each vector. Consistent X-gal staining was observed in the developing eye, specifically in the POM, only in the embryos injected with the vector comprising the WT ‘i8’. There was no consistent expression of mutant ‘i8’ (Fig. 4 and Sup. Fig. 5) or of wild type ‘i9’ (Sup. Fig. 5) in the embryonic eyes. However, in one of the mutant-‘i8’ embryos we noticed a mild phenotype (weak lacZ staining) consistent with the WT ‘i8’ phenotype, pointing out that the variant may not abolish the enhancer properties altogether.

## Discussion

We studied two consanguineous pedigrees of Jewish Iranian descent, with apparently recessive heredity of severe ocular malformations within the MAC continuum. Most of the affected individuals were born with one or two eyes being either absent or very small, malformed and almost blind, and thus suffered life-long profound visual and cosmetic disabilities. Using SNP microarray-based linkage analysis, we found in pedigree one a shared locus of ~1.5 Mbp on chromosome 2q35. Homozygosity mapping in pedigree 2 identified several possible disease-associated loci, one of which overlapped with the locus identified in pedigree 1: a shared segment of ~0.5 Mbp on chromosome 2q35 consisting of the same homozygous haplotype (combined maximal LOD score 6.8). Trio whole genome sequencing focusing on this locus identified 13 homozygous rare single nucleotide variants and small indels, only one of which, an intronic variant in *NHEJ1*, was not found in gnomAD database and was evolutionary conserved (phyloP *p*-value of 0.000001403). This variant was not found in a screen of ethnically matched controls and segregated with the phenotype in both pedigrees.

The variant was predicted not to affect NHEJ1 protein. Therefore, a regulatory role of a biologically relevant neighboring gene, *Ihh*, was considered. In mice, *Ihh* null mutant embryos have focal loss of the sclera in the posterior eye segment (optic nerve head region) and abnormalities in the choriocapillaris; their RPE has multiple foci of hypopigmented spots, with a high incidence of retinal detachment^18,20^. Although the eye sizes of *Ihh* null mutants were comparable to their wild-type littermates, their shape was described as “cauliflower-like”^18^. Those phenotypes resemble the CEA canine Collie phenotype^26^ and the human phenotype we describe, which are similar, with coloboma in the optic nerve head or its region, chorioretinal dysplasia, and retinal detachment.

Bioinformatics analysis demonstrated that the intronic NHEJ1 variant was within the human orthologous sequence of an experimentally verified limb-development enhancer of the mouse *Ihh*, termed “i8”: while there were no previous reports of *Ihh* enhancers acting in eye development, an in vivo enhancer-reporter assay demonstrated *Ihh*-like temporal and spatial expression for a reporter (lacZ) gene attached to this enhancer in the limb bud^16^. Additional findings supported a regulatory role of i8 in humans. In several human cell lines of blood and liver lineages, the specific conserved region of’i8‘ was predicted to be an active regulatory element: it is marked with H3K27ac, H3K4me1, highly sensitive to DNaseI, and is a validated binding site for several transcription factors (Fig. 2a and Sup. Fig. 3). The cell line data are in line with Dakubo et al.‘s findings that the *Ihh* signaling, which is critical in normal eye development of mouse embryos, originates from the choroid-endothelial cells^18^. Also of relevance were prior studies of a canine phenotype of choroidal hypoplasia similar to human coloboma, which identified a disease-associated 7.8k base pair deletion within the same intron in the dog *NHEJ1* ortholog^23^. The deletion in dogs erases the neighboring CNE, another verified component of the multipartite enhancer of *Ihh* termed’i9‘^16^.

First, we demonstrated in a chick model that the orthologous chicken ‘i8’ enhancer indeed acts as a regulator in eye development. Once this preliminary evidence was established, we went on to the mouse model, closer resembling human eye development. Using the in vivo enhancer assay applied by Will et al.^16^, we examined possible eye activity of enhancers’i8‘ and’i9‘, along with an ‘i8’ enhancer in which we inserted the human variant in the mouse ortholog. We found that adding the wildtype sequence of ‘i8’ to a minimal reporter promoter was consistently sufficient to drive eye expression in transgenic mice during embryogenesis at E11.5. In contrast, the mutated version of this sequence, with the same single-nucleotide variant found in our patients, did not consistently drive eye expression in transgenic mice during embryogenesis at E11.5.

The in vivo transgenic enhancer assay depends on undirected integration of the enhancer–reporter sequence into the genome of the transgenic embryo^27–29^. As each insertion event is independent, consistent reporter expression pattern is required (at least 3 embryos out of 5–10 embryos) in order to consider a DNA element as a positive enhancer^27–29^. Thus, the inconsistent activation driven in the eyes by the mutated’i8‘ in the minority of the cases could be attributed to the copy number and the integration sites (i.e., positional effect) that can cause ectopic domains of expression^27,29^.

Interestingly, the variable expression of the phenotype in the patients (Table 1) could be associated with variable *Ihh* deficiency caused by disruption of *Ihh* enhancer activity. The expression level of *Ihh*, especially in the POM and RPE of the developing eye, is essential for normal eye development^18,19,30^, indicating the importance of spatiotemporal transcriptional regulation of *Ihh*. Therefore, it is possible that patients with the ‘i8’ enhancer variant have variable Ihh expression levels, which might explain their variable phenotype. Given the recessive inheritance pattern, position of the i8 enhancer relative to the Ihh transcription start site, functional enhancer data, and overall parsimony, a loss-of-function enhancer mechanism seems most likely. However, the possibility that the mutation exerts gain-of-function with recessive inheritance, as reported for alpha thalassemia^31^, cannot be ruled out. Interestingly, the mouse ‘I8’ enhancer drives strong Ihh expression in the limbs, digits, growth plate and skull, and skeletal malformations (syndactyly, craniosynostosis) have been noted in humans with segmental duplications spanning the i8 ortholog^16^. Nevertheless, none of the affected individuals in our cohort had skeletal or cranial abnormalities, possibly due to functional redundancy with other elements such as i1, i5, i6, i7 and/or i8^16^.

Notably, the 3108 bp sequence of the human ‘i8’ enhancer is 40.2% identical (63.8% of span) to that of its chicken ortholog, 88.6% identical (100.0% of span) to its dog ortholog, and 81.8% identical (100.0% of span) to its mouse ortholog, and all three orthologs share the exact core “GGGTGA” sequence mutated in the MAC patients (Fig. 2b, c). The mutated ‘i8’ enhancer’s loss of activity further demonstrates that the molecular cause of the disease is the dysregulation of the enhancer’s target, *IHH*. The question that remains is what mediates the negative effect of the single-nucleotide variant in the ‘i8’ enhancer. Enhancers contain multiple binding sites for different transcription factors (TFs)^29^. In recent years, several cases of genetic diseases caused by non-coding mutations that disrupt transcription factor motifs in regulatory elements have been identified^2,32^. In some, proof that enhancer activity was decreased due to alteration of a highly conserved nucleotide in a transcription factor binding site was obtained^31,33,34^. The variant described in this study falls in a rVista - predicted binding site of PAX4, an important paralog of PAX6, the “master regulator” of eye and central nervous system morphogenesis^35^. This is further supported by our ATAC-sequencing data in RPE cells within the developing chicken. PAX4 is known to compete with PAX6 (UniProtKB - O43316), and while such competition has not been specifically demonstrated in eye development, it is plausible to have an effect also here. The variant may attenuate the binding and the effect of several possible TFs (PAX4 and others depicted in Sup. Fig. 3). However, as PAX4 is known to be expressed in the pancreas and in mature photoreceptors, but not in relevant segments of the developing eye, a role of altered PAX4 binding is questionable. Possible binding to PAX6 should be considered, as PAX6 expression in the developing eye is more relevant to the phenotype^36^. Although variable, depending on partner TFs, which may bind cooperatively, the core of the original consensus PAX6 paired domain (PD) site is TTCACGC^37^. The i8 sequence TTCACcC is mutated in the patients to TTCGCcC. The affected base is relatively invariant in the PAX6 position weight matrices (PWM). In fact, among mouse, dog, human, and chicken, the core bindings site is YTCACCC, further suggesting relevance of altered PAX6 binding to the mutated sequence^36^. Further studies are needed in order to determine which transcription factors may be relevant and active in modulating i8 enhancer activity in eye development. Other possibilities, such as altering the nucleotide composition of motif-neighboring sequences, the chromatin context of other genuine binding sites, and the three-dimensional (3D) structural conformation of DNA, also exist^32,38^.

The ‘i9’ enhancer^16^, which is deleted in CEA-affected dogs^23^, includes what Parker et al. described as the “core” of the CEA-associated deletion^23^. Therefore, we expected to see evidence of ‘i9’ enhancer activity in the developing eye, as with ‘i8’. However, ‘i9’ failed to drive expression in the developing eyes of E11.5 transgenic mice. This might be due to possible dependence of ‘i9‘ on the presence of additional cis-regulatory elements, which were not included in our vectors. Secondly, it is plausible that’i9‘ is active only at time points other than the single time point (E11.5) assayed. Thirdly, a recent study demonstrated that dogs harboring the 7.8k base pair deletion do not necessarily demonstrate the canine disease phenotype, suggesting that this deletion in dogs might be a marker adjacent to the pathogenic variant rather than being causative of the phenotype^39^. In fact, Brown et al. raised further doubt regarding the causality of the deletion^40^. Further studies on CEA -affected and unaffected dogs of the same breed (Danish rough Collies) with the 7.8 kb intronic deletion^39^ might elucidate the actual genetic cause of this canine phenotype, possibly unraveling another variant in other adjacent *Ihh* enhancer(s) in this locus, as no coding variants were found in that locus^23^.

Finally, recent studies have shown that control of *Ihh* signaling is fundamental in choroid fissure closure also in zebrafish morphants^41^, where ocular coloboma was found to be due to increased expression of the Hh pathway ligand *Indian Hedgehog b (ihhb)*. Another study using zebrafish morphants found that the absence of *Indian Hedgehog a (ihha)* in zebrafish morphants led to smaller eyes^42^. The Zebrafish studies have the inherent problems of the existence of two *ihh* paralogs and the lack of evident orthologs of the ‘i8’ mouse enhancer. Nevertheless, those studies also point to several roles for *IHH* in the developing eyes, including choroid fissure closure and eye size.

In summary, we have identified and delineated the first *IHH* enhancer active in eye development and demonstrate that a single-nucleotide variant in this enhancer, residing within an intron of the neighboring gene, *NHEJ1*, perturbs the enhancer’s activity and causes autosomal recessive MAC in humans. Further studies are needed to elucidate the precise transcriptional pathogenic mechanisms downstream of the *IHH* enhancer mutation. Altogether, the findings broaden our understanding of the molecular mechanisms involved in ocular growth and development, as well as the role of non-coding mutations in human disease.

## Methods

### Ethics statement

All procedures were in accordance with all relevant ethical regulations for animal testing and research and the ethical standards of the institutional and the national research committee and with the 1964 Helsinki declaration and its later amendments or comparable ethical standards. Specifically, this study was approved by the Soroka Medical Center Institutional Review Board (IRB approval #5071G) and the Israel Ministry of Health National Helsinki Committee (approval #920100319).

### Clinical phenotyping

Affected individuals were examined by senior geneticist, pediatrician, and ophthalmologists. Patients underwent thorough ophthalmic examination, including testing of visual acuity, refractive errors, eye movements, ocular alignment, and examination of the anterior and posterior segment of the eye. For young patients, evaluation of vision was performed based on their fixation behavior, and the eye exam was performed under general anesthesia due to insufficient cooperation. A subset of affected individuals had ocular ultrasound exam and full field electroretinography.

### Genomic DNA extraction from whole blood or saliva

Blood or saliva samples were obtained following written informed consent from all individuals studied or their legal guardians. Blood samples (3–10 ml) were collected in BD™ EDTA tubes, and total genomic DNA was extracted using E.Z.N.A.® Blood DNA Kit. Saliva samples were collected in OG-500 Oragene™ Saliva DNA Collection Kit (Oragene™, Ottawa, Ontario, Canada) and genomic DNA was extracted per manufacturer’s instructions.

### Linkage analysis

Samples were derived from the 25 members of pedigrees P1 and P2 with marked genotypes (Fig. 1a). Genome-wide linkage analysis was performed as previously described^43^. In short, using Illumina GSAv2 (Infinium™ Global Screening Array v2.0; Illumina, San Diego, CA, USA) > 665 K markers per sample were genotyped. All informative SNPs were used for subsequent analyses. Homozygosity mapping for both pedigrees (separately and combined) was carried out using Homozygosity-Mapper^44^, using 309,811 markers spread across all autosomal chromosomes, and was set to retrieve all common homozygous regions shared by the seven available affected individuals, with no lower threshold set for the size of homozygosity loci. Regions of over 50 sequentially identical homozygous markers appearing in at least one healthy control were omitted. Next, multipoint LOD score for both pedigrees was calculated via SUPERLINK ONLINE SNP 1.1^45^ software, zooming into the locus found in the homozygosity mapping (using all segregating SNPs), in groups of 6 SNPs per window. Physical positions are of GRCh37/hg19 genome assembly.

### Trio whole genome sequences analysis

Whole Genome Sequencing (WGS) trio of the proband (P1:VI-2, Fig. 1a) and her parents were performed by the Novogene Corporation bioinformatics team (Palo Alto, CA, USA). In short, genomic DNA samples were fragmented to 350 bp inserts, and the libraries were generated using Truseq Nano DNA HT Sample Preparation Kit (Illumina USA). Sequencing was performed using HiSeq X (Illumina, San Diego, CA, USA) and paired-end 150-bp read protocol. The average percentage of Q30 was above 84% and the error rate below 0.05%. At least 90 Gb raw data per sample were obtained. Standard Novogene bioinformatics analysis included alignment with the reference genome (1000 Genomes GRCh37 + decoy human genome) and statistics of sequencing depth and coverage; SNP/InDel, SV and CNV calling, and variants annotation and statistics each using the relevant software (BWA, SAMtools, Picard; GATK, ANNOVAR; Delly, ANNOVAR; control-FREEC, and ANNOVAR in concordance). Data were next analyzed using QIAGEN’s Ingenuity Variant Analysis software (www.qiagen.com/ingenuity, QIAGEN, Redwood City, CA, USA), last accessed December 31, 2020 (Ingenuity Variant Analysis version 7.1.20201218). We excluded irrelevant common variants with an observed allele frequency of more than 1% in any of the public databases (in parentheses- version): 1000 Genomes project (phase3v5b), ExAC (0.3.1), gnomAD (2.1.1), and NHLBI ESP exomes (ESP6500SI-V2), or found in homozygous state in one or more of our in-house WGS controls. We kept all proband sample variants found on chromosome 2 between positions 219821659 and 220384126 (either interpreted as homozygous or heterozygous, without excluding low-confidence variants).

To ensure the reliability of the variants, IGV^46,47^ was used to assert the variants manually. Variants not truly homozygous in the proband sample and heterozygous in the parents were excluded. Beyond the automated analysis, the entire locus was carefully re-examined against other control genomes using IGV for other segregating and rare structural variants, including indels and possible copy number variations.

The variants were assessed and prioritized initially based on known pathogenic variants found in HGMD pro (v. 2020.3), ClinVar (2020-09-15), and OMIM (July 06, 2020). Next, they were assessed based on gnomAD frequency, gnomAD Jewish frequency, gnomAD homozygous count, 1000 Genomes frequency, in-house WGS homozygous count, and by conservation phyloP *p*-value (2009–11), CADD Score (v1.6), and Ingenuity’s computed ACMG guidelines classification.

### Segregation analysis and population screening

The NHEJ1 intronic variant was further assayed through Sanger sequencing using forward and reverse primers: [F:5’- aggaagctccttgcattgct-3’, R:5’- ataggtctggtggtaggggg-3]. We also applied restriction fragment length polymorphism (RFLP) analysis to both pedigrees and 87 ethnically matched controls, using forward and reverse primers: [F:5’- ggaagctccttgcattgctg-3’, R:5’- gctacctcggatgaggaaca -3’]. RFLP using the restriction endonuclease HphI (New England Biolabs, Ipswich, MA, USA) yielded 82 and 66 bp segments for the wildtype sequence compared with 148 bp for the mutant (uncut). Primers were designed using Primer-Blast^48^.

### Assessment of the *NHEJ1* variant

Prediction tools used included MutationTaster^49^ and Human Splicing Finder^50^. UCSC genome browser (http://genome.ucsc.edu) was used to study the human chromosome 2q35 locus and the relevant conserved non-coding elements in humans, dogs, chickens and mice, and to convert the genomic location between species (using “view- in other genomes” or retrieving the DNA and performing BLAT)^51,52^. NCBI’s BLAST (https://blast.ncbi.nlm.nih.gov/Blast.cgi) was used to align the DNA sequences, and for searching in the Zebrafish genome^53^. Regulatory tracks were presented on UCSC, based on ENCODE, GeneHancer (GeneHancer Hub at the UCSC Golden Path -directed from GeneCards)^54–56^.

### *Gallus gallus* ATAC-seq library preparation and sequencing

Specific-pathogen-free chicken eggs (Charles River Laboratories, catalog 10100329) were incubated in a humidified incubator at 38 °C and collected at embryonic day 4 or 5 (Hamburger Hamilton stage 24 and 26). Eyes were enucleated and washed in cold PBS, and a sheet of RPE from the posterior eye chamber was collected directly into cold ATAC-seq lysis buffer (Active Motif cat. 53150). The RPE from two embryos were collected per biological sample. Biological replicates were carried out in duplicate and ~100,000 nuclei were loaded per reaction. Library preparation was carried out per manufacturer’s instructions (Active Motif cat. 53150), and final libraries were validated on the Agilent Bioanalyzer before sequencing on a lane of HiSeq 4000. Each sample was sequenced to a minimum depth of 80 million 150 base pair paired end reads. Raw reads were quality trimmed using trim galore with the parameters –clip_R1 16 –clip_R2 18 –three_prime_clip_R1 6 –three_prime_clip_R2 4^57,58^ and high-quality reads were aligned to the chicken genome (GRCg6a) using Bowtie 2^59^ with the parameters –very-sensitive -k 5 -p 40^59^. Biological replicates were collapsed and visualized using Integrative Genomics Viewer^46^.

### Plasmid constructs

The ptkEGFP_v2 and ptkmCherry_v2 backbones were developed and shared by Masanori Uchikawa, as previously described^60^. Hsp68mp-lacZ backbone was taken from previous studies^4^. High fidelity Q5® polymerase (New England Biolabs, Ipswich, MA, USA) was used for all PCRs, including insertion of deferent restriction enzymes’ recognition sites in the inserts’ borders and site-directed mutagenesis using the primers appearing below. The Cloning Kit of pJet (Thermo Scientific CloneJET PCR Cloning Kit) and basic cut & paste (sub)cloning were used to attain all constructs (Sup. Table 2). Cloning design and sequence analyses were done using SnapGene software (Insightful Science; snapgene.com). The plasmids for enhancer assays in chicken and mice embryos are based on a minimal promoter that activates the reporting gene only upon insertion of specific tissue-dependent enhancers.

Generation of the constructs for the chicken experiments (“gal-i8-ptkEGFP ver2” and “gal-i8-ptkmCherry ver2”): the genomic region (putative enhancer) of Chicken ‘i8’ was amplified from DT40 extracted DNA (Chicken bursal lymphoma cell line, ATCC) genome using primers 5’-GGTAccggaggagcccaagcacaaat-3’ & 5’- CTCGAGctttctgccctttcactgcc-3’ and inserted into pJet (Thermo Scientific CloneJET PCR Cloning Kit) to be subcloned into the “ptkEGFP_v2” and “ptkmCherry_v2” backbones. Generation of the constructs for the mouse experiments: the two genomic regions (putative enhancers), Mouse “i8” and “i9”, were amplified from the C57BL/6 mouse genome, using primers 5’-ttgaggcagaaggattgtcata-3’ & 5’-agccagaggtcaacatttgagt-3’ (for “mice i8”), and 5’-gctgagatgaatgacagtgagg-3’ & 5’-gtcacacctgatgatctgcatt-3’ (for “mice i9”), as described in Will et al.^16^, and inserted into blunt cut-open hsp68mp-lacZ^4^ (to create “mice_i8-Hsp68mp-lacZ”, and “mice_i9-Hsp68mp-lacZ”, accordingly). Site-directed mutagenesis, introducing the human *NHEJ1* variant (NM_024782.2): c.588+18131 A > G (or NG_007880.1: g.37317 A > G) to the mouse sequences, was done using primers 5’- atggggCgaagaggagggcaggaattg-3’ and 5’- ctcttcGccccatacagctaggaattagtgg-3’ (for “mice_i8-MUT-Hsp68mp-lacZ”). All plasmids were validated by Sanger sequencing. Plasmids were transformed into competent *E-coli* and purified using the Presto™ Mini Plasmid Kit (Geneaid, New Taipei City, Taiwan). Large preparations for chicken embryos injections were done using Geneaid Midi/Maxi Plasmid Kit, Endotoxin Free (Geneaid, New Taipei City, Taiwan).

### Transgenic chicken embryos enhancer-reporter assay

The transgenic chicken embryos were generated as previously described (https://bio-protocol.org/e1498)^61^. Briefly, the Chicken ‘i8’ putative enhancer construct (“gal-i8-ptkEGFP ver2” or “gal-i8-ptkmCherry ver2”) was concentrated to 1–2 ug/µl and injected into the optic vesicles of chicken embryos at Hamburger-Hamilton stage 9–12. Injections were performed using borosilicate capillary tubing for injection (FHC, catalog. 30-30-1) made with a micropipette puller. An electrolyte solution of 100 µl of HHBS or Ringer’s Solution was added to embryos before electroporating with an ECM 830 High Throughput Electroporation System using the following settings: 18 V, 50 ms pulse length and 3 pulses, as previously described^61^. Electroporation was performed with platinum/iridium microelectrodes designed to the previously described dimensions^61^. Fluorescence was observed 2 and 3-days post-electroporation and visualized on green channel using the Zeiss Discovery V8 and V12 SteREO Microscopes (Carl Zeiss Microscopy GmbH, Jena, Germany). In all cases throughout the study, electroporation of a control ptkEGFP enhancer construct in the absence of the i8 promoter did not result in any observable fluorescence.

### Transgenic mouse enhancer-reporter assay

Mouse enhancer assays were carried out in transgenic mouse embryos as previously described^4^, performed by Cyagen Biosciences (Cyagen US inc.), whose facility meets animal health and welfare guidelines. In short, following validation of the ‘mice embryos’ vectors (mice_i8-Hsp68mp-lacZ, mice_i8-MUT-Hsp68mp-lacZ, mice_i9-Hsp68mp-lacZ, and a negative control- Hsp68mp-lacZ without a putative enhancer), they were bacterial-amplified and purified, linearized and pronuclear injected to C57BL/6NxC57BL/6N mice fertilized oocytes. Oocytes were then transferred into the oviducts of pseudo-pregnant mice. Embryos were retrieved from surrogate mothers at E11.5, genotyped by PCR (primer sequences available upon request), fixed in 4% paraformaldehyde, and X-gal stained for the expression of LacZ in the embryos at E11.5. Empty vector (Hsp68mp-lacZ)-injected embryos served as negative control. Enhancers showing consistent reporter gene expression in the relevant tissue among at least three embryos were defined as positive; putative enhancers assayed were defined as negative (for this embryonic day only) when no reproducible pattern was observed among a minimum of five transgenic, PCR positive and lacZ positive embryos (per x-gal staining in the embryos)^27^.

### Histology studies

Coronal plane sections of the transgenic (and X-Gal stained) mouse embryos’ eyes were obtained by Excalibur Pathology Inc (Norman, OK, USA). Processing included paraffin embedding, sectioning and nuclear fast-red counterstaining.

### Reporting summary

Further information on research design is available in the Nature Research Reporting Summary linked to this article.

### Supplementary information

Supplemental Material

Reporting Summary

## Data Availability

Gallus gallus ATAC-seq data have been deposited in NCBI’s Gene Expression Omnibus and are accessible through GEO Series accession number GSE197935^62^. The variant, *NHEJ1* (NM_024782.2): c.588+18131A > G (NG_007880.1: g.37317A > G), was submitted to ClinVar (SCV002769719). Note that NGS data public sharing was not included in the informed consent signed by the patients. Other data generated or analyzed during this study are included in this published article [and its supplementary information files], additional data is available from the corresponding author on reasonable request.

## Code availability

Any code used in this study is available upon request to the authors, with no restriction to access. Parameters for the employed software are detailed in methods.
