# Supplementary material for: IHH enhancer variant within neighboring NHEJ1 intron causes microphthalmia anophthalmia and coloboma
Source: NPJ Genom Med. 2023 Aug 14;8:22. doi: 10.1038/s41525-023-00364-x (PMC10425348; doi:10.1038/s41525-023-00364-x)
Supplement: Supplementary file 1 — Supplemental Material [file 41525_2023_364_MOESM1_ESM.pdf]

Supplemental material:

IHH enhancer variant within neighboring NHEJ1 intron causes microphthalmia, anophthalmia and coloboma

Ohad Wormser*, Yonatan Perez*, Vadim Dolgin*, Bahman Kamali, Jared A Tangeman, Libe Gradstein, Yuval Yogev, Noam Hadar, Ofek Freund, Max Drabkin, Daniel Halperin, Inbar Irron, Erika Grajales-Esquivel, Katia Del Rio-Tsonis, Ramon Y. Birnbaum, Gidon Akler‡, Ohad S. Birk‡.

* -Contributed equally.

‡ Corresponding authors

Sup. Fig. 1. Genome-wide homozygosity and LOD score in Iranian Jews:
In pedigree 1 (P1, Fig.1a), a single homozygous stretch was found on chromosome 2, that was shared by all available affected individuals and not by unaffected family members (individuals tested are marked with their genotypes in Fig. 1a). The maximal LOD score for this locus at chromosome 2 was 6.2153 at rs13016645 - rs6719949 (multipoint analysis, pedigree 1 only). Note that the true common homozygous locus in this pedigree starts at rs4552182 (at chr2: 219184872 build 37), as a single clearly segregating SNP can be used to further restrict the locus (red arrow). Hence, the true homozygous locus extends from rs4552182 to rs12694490, culminating in a ~1.5 million bp locus on chromosome 2q35. This locus is encompassed by a green dashed rectangle in the combined plots.
In pedigree 2 (P2, Fig.1a), both affected offspring were also found to harbor a shared homozygous stretch on chromosome 2q35, which they inherited from their heterozygous parents. This locus is encompassed in a yellow dashed rectangle in the combined plots. Crossing the two (the combined plot), a single shared genotype for all affected individuals was found between markers rs6436102- rs746233: a stretch of 39 markers homozygous to the same haplotype was found between those two markers (see the red rectangle in the combined plot, also zoomed-in on Fig.1c), corresponding to chr2:219821659-220384126 (GRCh37). The combined maximal LOD score for this 0.5 Mbp locus was 6.8169 at rs6753739 (multipoint analysis, both analyzed pedigrees combined).

- ***Pedigree 1:***

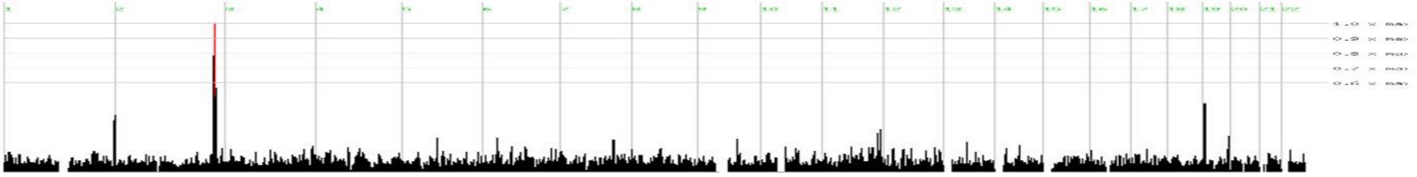

score	chr	from (bp)	to (bp)	from SNP	to SNP	build 37	
narrow - use this when all patients are in the same family							
1242	2	218883186	220733584	rs7580997	rs12694490	region	genotypes

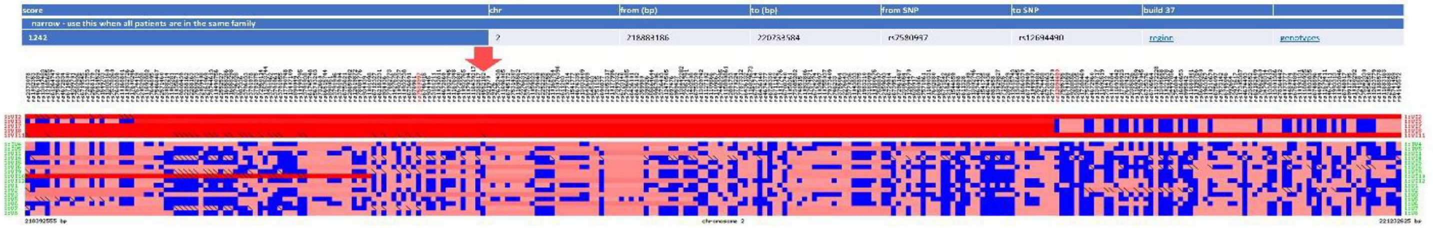

- ***Pedigree 2:***

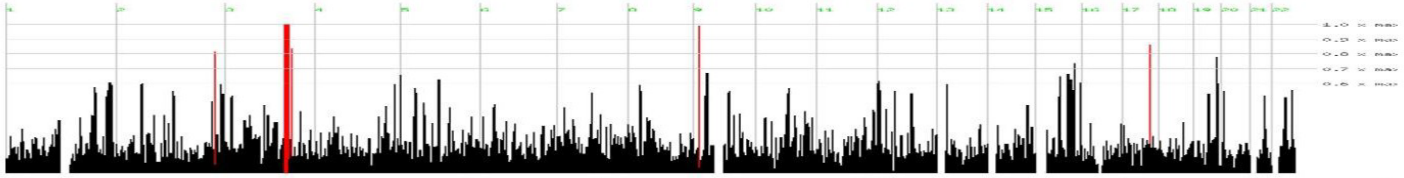

score	chr	from (bp)	to (bp)	from SNP	to SNP	build 37	
narrow - use this when all patients are in the same family							
300	3	132366498	141410864	rs6776500	rs4683620	region	genotypes
296	9	14079067	14838860	rs480700	rs2779509	region	genotypes
258	17	61785904	63495631	rs2013288	rs9892932	region	genotypes
250	3	147496495	147956156	rs4681375	rs1874256	region	genotypes
244	2	215821655	220926429	rs6436102	rs4674493	region	genotypes

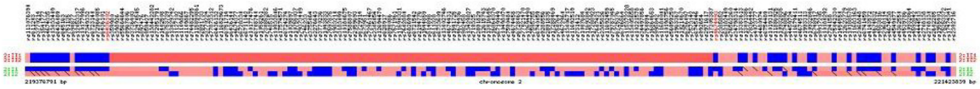

- ***Combined:***

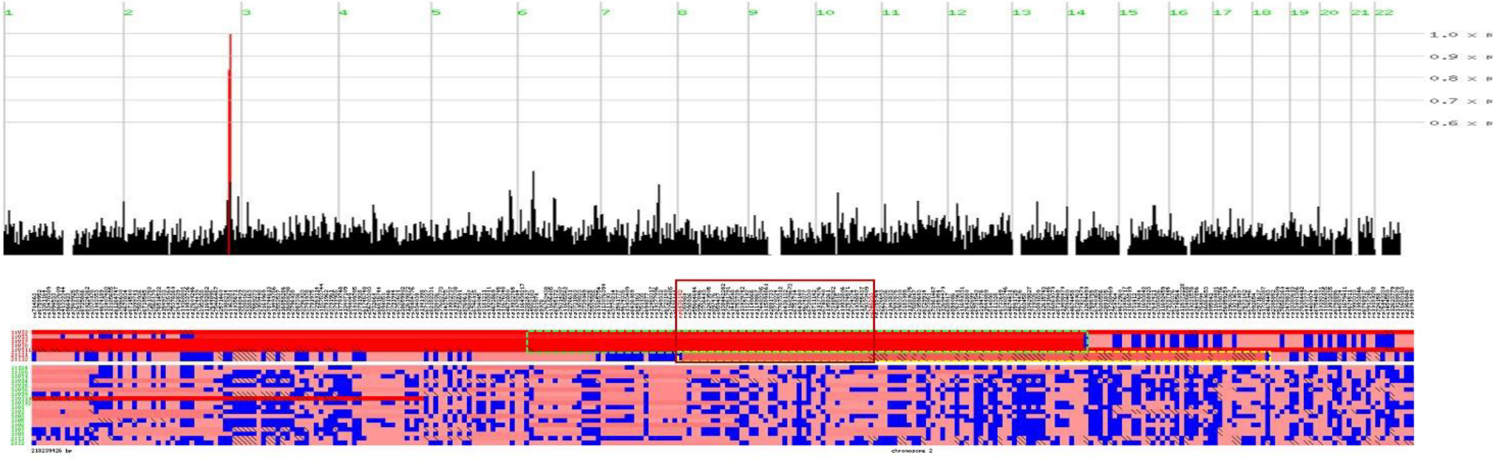

- ***Results of Single Locus MultiPoint Analysis for Project (zoomed around locus at chromosome_02, same boundaries as the above homozygosity mapper genotype view subfigure)***

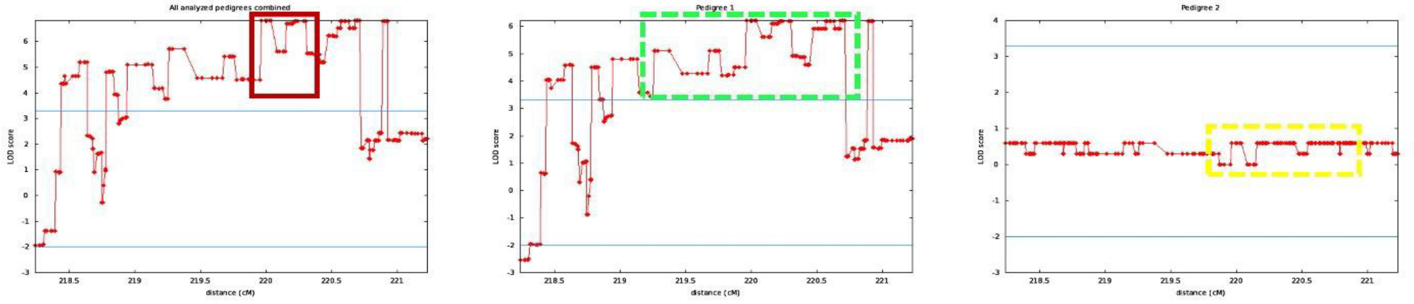

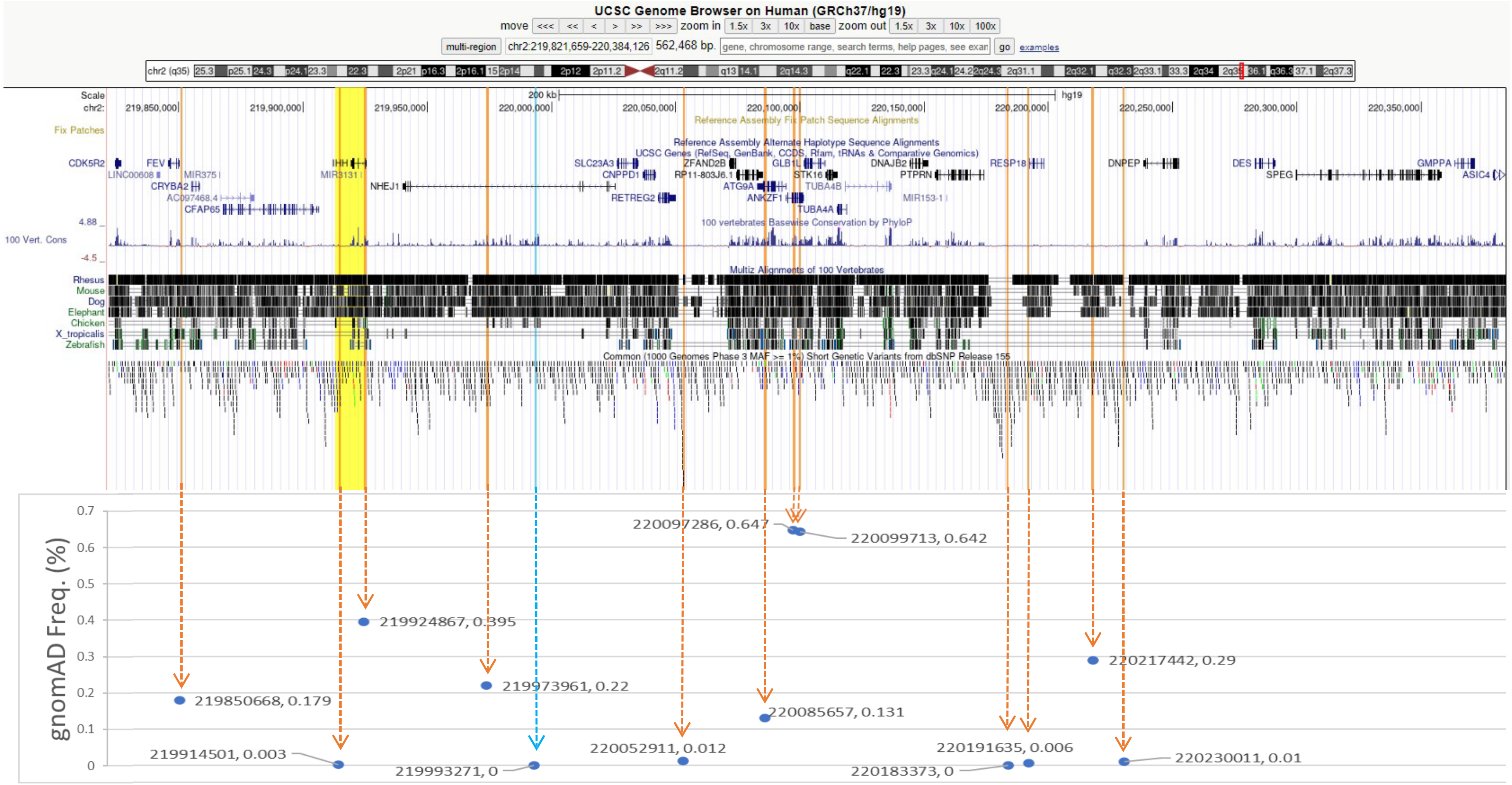

Sup. Fig 2. Genomic view of the disease-associated 0.5 Mb homozygous chromosomal locus.
The human disease-associated locus was mapped to chr2:219821659 - 220384126 bp. All the variants identified within the locus (from Sup. Table 1) are depicted in orange, except for the “i8” enhancer mutation which is depicted in light blue (as in main figure 2). In yellow- the Chicken chr7:21798705-21810600 locus (Nov. 2011; ICGSC Gallus_gallus-4.0/galGal4), an 11,896 bp segment, whose deletion was identified as the causative mutation underlying the semi-lethal Creeper trait (Jin, S. et al. Deletion of Indian hedgehog gene causes dominant semi-lethal Creeper trait in Chicken. Sci. Rep. 6, 30172; DOI: 10.1038/srep30172 (2016)). Using UCSC's "View- in other genomes" tool, the genomic locus orthologous to that of the Creeper trait-associated deletion in chicken was mapped to human chr2:219912908-219924684 (identity - 24.5% of bases, 72.6% of span). The lowermost panel shows for each of the variants their position and gnomaAD frequency, in percentages, as follows: position on chromosome 2, frequency. All human genomic sequences are per Feb. 2009 (GRCh37/hg19

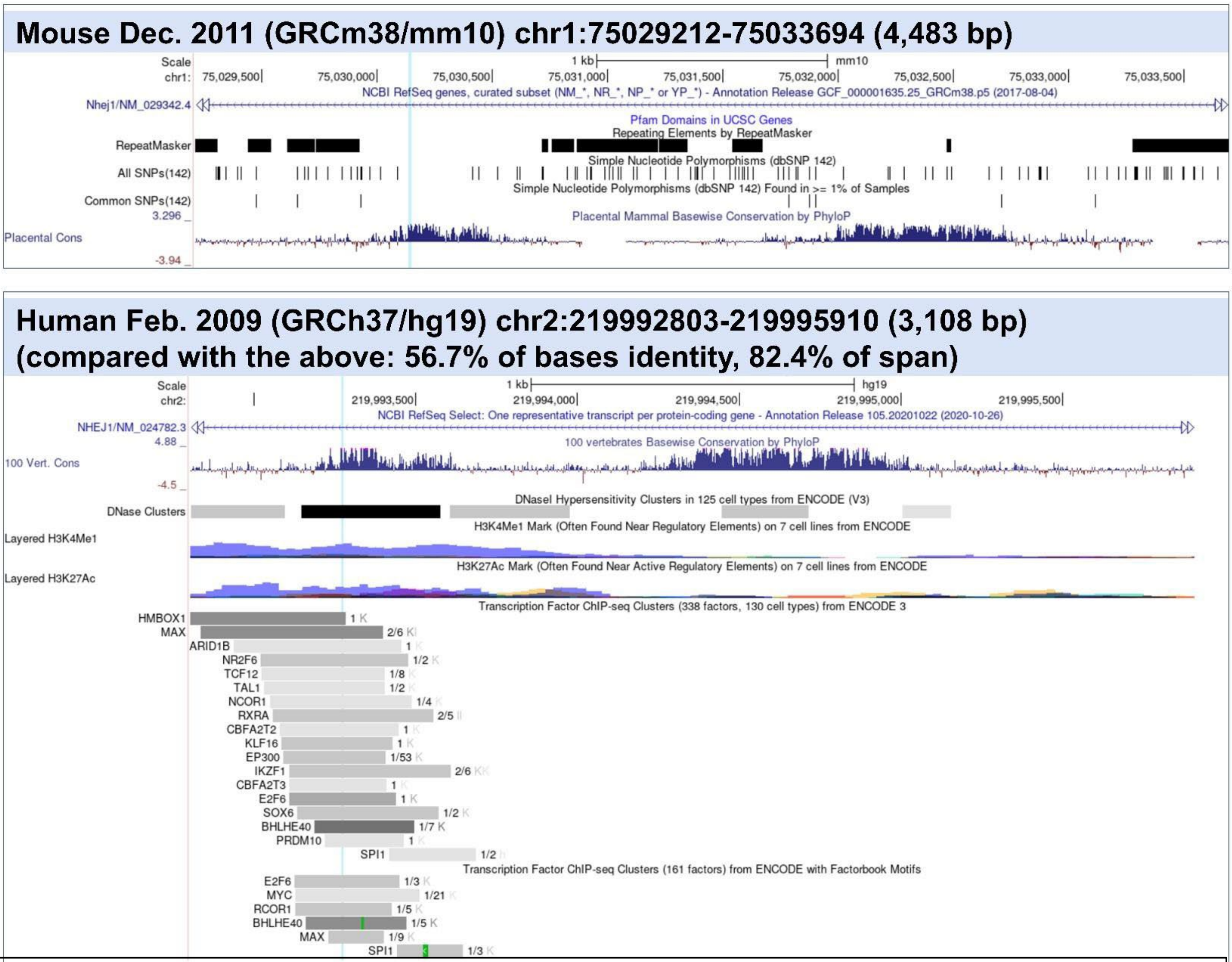

Sup. Fig. 3. Genomic view of the "i8" validated *Ihh* enhancer in humans and mice: Mouse verified 'i8' enhancer (Will et al., 2017) converted to Human hg19 using 'view- in other genome' (UCSC), presented along with transcription factors, DNaseI, H3K4me1 and H3K27ac marks. In light blue - the variant found in the present study. H3K4me1 and H3K27ac marks (often found near regulatory elements) on K562 cells are presented in purple (data derived from ENCODE). 'K' (appearing in the transcription factor tracks)- stands for K562 cell line, where 'l' stands for liver cells, and 'h' for HL-60 cells. Last accessed March 2022.

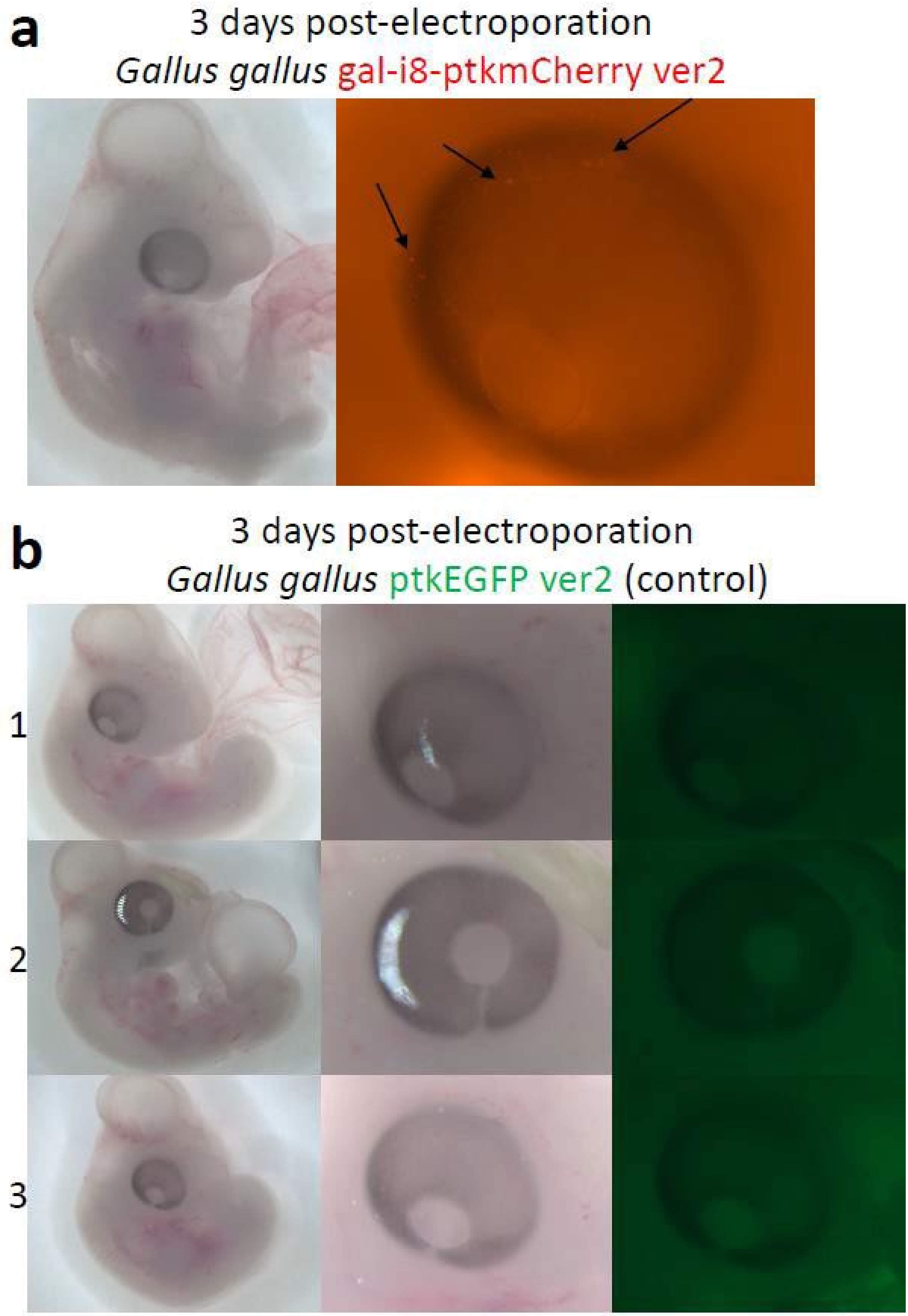

Sup. Fig. 4. Representative embryos from the control group. (a): Fluorescence observed across the ocular surface of an embryo electroporated with the gal-i8-ptkmCherry ver2 at embryonic day 2 and visualized 3 days later. (b) Three representative embryos are shown following electroporation with the gal-i8-ptkEGFP ver2 control plasmid and imaged. During the study, no fluorescence was detected in embryos receiving the plasmid without the i8 enhancer (n=19).

Putative enhancer ‘i9’: (inconsistent with *Ihh* expression)

a

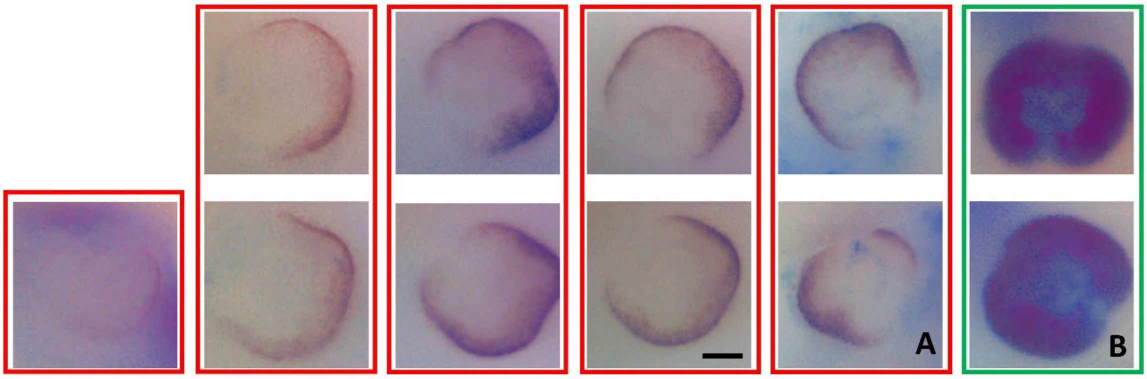

b

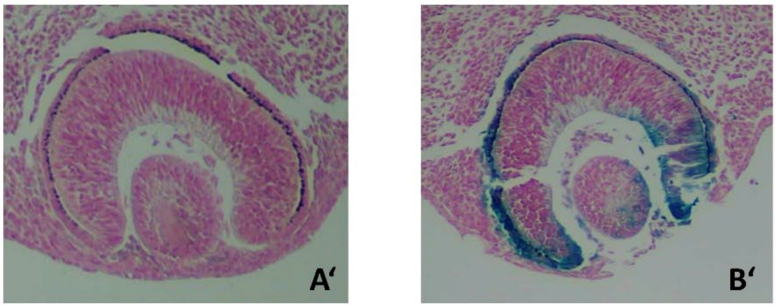

Mutated ‘i8’ (omitted artifact)

c

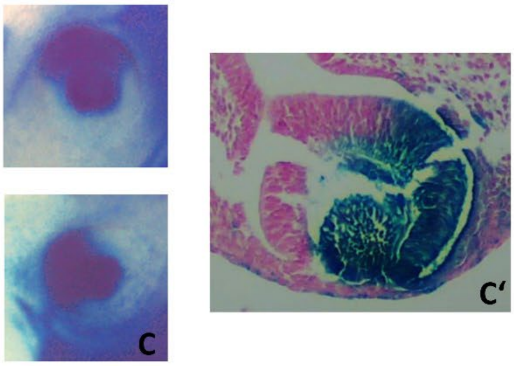

Sup. Fig. 5. WT 'i9' Ihh enhancer activity does not phenocopy *Ihh* eye expression in mouse embryos, and an omitted artifact. In vivo enhancer-reporter assay was applied to compare with the known expression pattern of Ihh in the developing eyes of embryonic day (E) 11-12d mouse embryos (G.D. Dakubo et al., 2008). Three putative Ihh enhancers were cloned and inserted into Hsp68 minimal promoter - lacZ reporter vectors: two previously identified limb Ihh's enhancers of the mouse genome (termed 'i8' and 'i9' by Will et al., 2017), and a mutated version of 'i8' (site-directed mutagenized to contain the exact point variant found in the patients). A negative control vector, without a putative enhancer, was also tested (see main figure 4). **Panel A** - all eyes of the ‘i9’; upper and lower images in each rectangle represent both eyes of the same samples, when available. The X-Gal staining in both eyes in the rightmost sample is positive although not POM specific. No consistent X-gal staining (more than 3 of at least five embryos) was noted. **Panel B** – a’ and b’ depict coronal sections (nuclear fast red counterstain) of a and b above. **Panel C**- an omitted sample from the Mutated ‘i8’ series, although it has positive expression- the X-Gal staining in both eyes is not anatomically localized, suggesting an artifact, thereby not considered. The scale bar represents 0.1mm.

Chr.	Position	Ref.	Alt.	Gene Region	Gene Symbol	Transcript Variant	Locus	gnomAD Freq.	gnomAD Jewish Freq.	gnomAD Homozygous Count	Conservation phyloP p-value	CADD Score	Classification (automated)	dbSNP ID	Interpretation:
**2**	**219993271**	**T**	**C**	**SNV**	**NHEJ1**	**c.588+18131 A>G**	**+**				**0.000001403**	**18.68**	**Uncertain Significance**		**Best candidate**
2	220183373		TTTT TTTT	Insertion			+						Uncertain Significance		Not conserved, Low complexity region, multiallelic (see rs1198661897)
2	219914501	G	A	SNV			+	0.003	0	0		< 10	Uncertain Significance	956556048	Not conserved
2	220191635	C	A	SNV			+	0.006	0	0		< 10	Uncertain Significance	758976220	Not conserved
2	220230011	C	A	SNV			+	0.01	0	0		< 10	Uncertain Significance	1342306857	Not conserved
2	220052911	T	C	SNV			+	0.012	0	0		< 10	Uncertain Significance	62191826	Not conserved
2	220217442	T	C	SNV			+	0.29	0	0		< 10	Uncertain Significance	148222797	Too frequent (Latino -0.590%), not highly conserved
2	219850668	C	T	SNV	FEV	c.-871G>A	+	0.179	1.724	0		< 10	Likely Benign	182542836	Too frequent among Jewish and low conservation
2	219973961	GG		Deletion	NHEJ1	c.589-31034_589-31033delCC	+	0.22	1.379	0		17.45	Uncertain Significance	559289909	Too frequent among Jewish and low conservation
2	220085657	C	T	SNV	ABCB6;<br>ATG9A	c.-2262G>A;<br>c.2369-43G>A	+	0.131	1.996	1		< 10	Likely Benign	200850274	Too frequent among Jewish and low conservation
2	219924867	G	A	SNV	IHH;<br>MIR3131	c.315+8C>T;<br>n.-1395C>T	+	0.395	2.297	5	0.00191	17.77	Likely Benign	186249490	Too frequent with 5 Homozygous, also benign according to ClinVar (see RCV000381108.1; RCV000309515.2; RCV000961119.2)
2	220099713	C	T	SNV	ANKZF1	c.1370C>T	+	0.642	2.085	13		15.22	Likely Benign	140395841	Benign (see also ClinVar RCV000961121.1)
2	220097286	G	A	SNV	ANKZF1	c.439G>A	+	0.647	2.085	13		19.21	Likely Benign	200506719	Benign (see also ClinVar RCV000961120.1)

Sup. Table 1. Prioritized variants from the genome sequences - trio (VI-2 vs V-5 & V-6) within the shared homozygous locus. Chr.- Chromosome, Ref.- Reference Allele, Alt.- Sample Allele, SNV.- single nucleotide variant, Freq.- Frequency (in percentages), Locus borders- chr2:219821659 - 220384126 bp. All locations according to hg19/GRCh37. Reference sequences: NHEJ1- NM_024782.3; FEV- NM_017521.3; ABCB6- NM_005689.4; ATG9A- NM_001077198.3; IHH- NM_002181.4; MIR3131- NR_036081.1; ANKZF1- NM_018089.3.

	Purpose	Plasmid full name	Putative enhancer sequence origin	Minimal promoter	Reporter	WT/ Mutant enhancer sequence	Notes
**1.**	Plasmids for enhancer assays in chicken embryos	ptkEGFP ver2	No putative enhancer	ptk	EGFP	Neither	Negative control (doi: 10.1007/978-1-4939-7216-6_12.)
**2.**		gal-i8-ptkEGFP ver2	Gallus gallus (galGal6_ chr7: 22278164-22279802)			WT	Chicken 'i8'
**3.**		gal-i8-ptkmCherry ver2			mCherry		
**4.**	Plasmids for enhancer assays in mouse embryos	Hsp68mp-lacZ	No putative enhancer	Hsp68	lacZ	Neither	Negative control
**5.**		mice _i8-Hsp68mp-lacZ	Mus musculus, AC104542			WT	Re-creation of Will's et al (doi: 10.1038/ng.3939.) plasmid (with mouse 'i8' putative enhancer)
**6.**		mice_ i8-MUT-Hsp68mp-lacZ				Mutant	Mice 'i8' with the human variant
**7.**		mice_i9-Hsp68mp-lacZ				WT	Re-creation of Will's et al (doi: 10.1038/ng.3939.) plasmid with mouse 'i9' (the enhancer deleted in CEA dogs)

Sup. Table 2: Plasmids for enhancer assays in chicken and mice embryos
