# Supplementary material for: IHH enhancer variant within neighboring NHEJ1 intron causes microphthalmia anophthalmia and coloboma
Source: NPJ Genom Med. 2023 Aug 14;8:22. doi: 10.1038/s41525-023-00364-x (PMC10425348; doi:10.1038/s41525-023-00364-x)
Supplement: Supplementary file 2 — Reporting Summary [file 41525_2023_364_MOESM2_ESM.pdf]

nature portfolio

Corresponding author(s): Ohad Birk

Last updated by author(s): Jun 22, 2023

Reporting Summary

Nature Portfolio wishes to improve the reproducibility of the work that we publish. This form provides structure for consistency and transparency in reporting. For further information on Nature Portfolio policies, see our Editorial Policies and the Editorial Policy Checklist.

Statistics

For all statistical analyses, confirm that the following items are present in the figure legend, table legend, main text, or Methods section.

n/a	Confirmed	
☒	☐	The exact sample size (*n*) for each experimental group/condition, given as a discrete number and unit of measurement
☒	☐	A statement on whether measurements were taken from distinct samples or whether the same sample was measured repeatedly
☒	☐	The statistical test(s) used AND whether they are one- or two-sided<br>*Only common tests should be described solely by name; describe more complex techniques in the Methods section.*
☒	☐	A description of all covariates tested
☒	☐	A description of any assumptions or corrections, such as tests of normality and adjustment for multiple comparisons
☒	☐	A full description of the statistical parameters including central tendency (e.g. means) or other basic estimates (e.g. regression coefficient) AND variation (e.g. standard deviation) or associated estimates of uncertainty (e.g. confidence intervals)
☒	☐	For null hypothesis testing, the test statistic (e.g. *F*, *t*, *r*) with confidence intervals, effect sizes, degrees of freedom and *P* value noted<br>*Give P values as exact values whenever suitable.*
☒	☐	For Bayesian analysis, information on the choice of priors and Markov chain Monte Carlo settings
☒	☐	For hierarchical and complex designs, identification of the appropriate level for tests and full reporting of outcomes
☒	☐	Estimates of effect sizes (e.g. Cohen's *d*, Pearson's *r*), indicating how they were calculated

Our web collection on statistics for biologists contains articles on many of the points above.

Software and code

Policy information about availability of computer code

Data collection	*Provide a description of all commercial, open source and custom code used to collect the data in this study, specifying the version used OR state that no software was used.*
Data analysis	Any code used in this study is available upon request to the authors, with no restriction to access. Parameters for the employed software are detailed in the methods section of the manuscript.

For manuscripts utilizing custom algorithms or software that are central to the research but not yet described in published literature, software must be made available to editors and reviewers. We strongly encourage code deposition in a community repository (e.g. GitHub). See the Nature Portfolio guidelines for submitting code & software for further information.

Data

Policy information about availability of data

All manuscripts must include a data availability statement. This statement should provide the following information, where applicable:

- Accession codes, unique identifiers, or web links for publicly available datasets
- A description of any restrictions on data availability
- For clinical datasets or third party data, please ensure that the statement adheres to our policy

Gallus gallus ATAC-seq data have been deposited in NCBI's Gene Expression Omnibus and are accessible through GEO Series accession number GSE197935 (https://www.ncbi.nlm.nih.gov/geo/query/acc.cgi?acc=GSE197935) 63. The variant, NHEJ1 (NM_024782.2): c.588+18131A>G (NG_007880.1: g.37317A>G), was submitted

to ClinVar (SCV002769719). Note that NGS data public sharing was not included in the informed consent signed by the patients. Other data generated or analyzed during this study are included in this published article [and its supplementary information files], additional data is available from the corresponding author on reasonable request.

Human research participants

Policy information about studies involving human research participants and Sex and Gender in Research.

Reporting on sex and gender	Seven individuals, of 2 large families, mixed sex, age- 1 to 30. Blood or saliva samples were obtained following written informed consent from all individuals studied or their legal guardians.
Population characteristics	Iranian Jews (originating from the vicinity of Mashhad in north Persia).
Recruitment	Both families approached us.
Ethics oversight	All procedures were in accordance with the ethical standards of the institutional and the national research committee and with the 1964 Helsinki declaration and its later amendments or comparable ethical standards. Specifically, this study was approved by the Soroka Medical Center Institutional Review Board (IRB approval #5071G) and the Israel Ministry of Health National Helsinki Committee.

Note that full information on the approval of the study protocol must also be provided in the manuscript.

Field-specific reporting

Please select the one below that is the best fit for your research. If you are not sure, read the appropriate sections before making your selection.

☒ Life sciences ☐ Behavioural & social sciences ☐ Ecological, evolutionary & environmental sciences

For a reference copy of the document with all sections, see nature.com/documents/nr-reporting-summary-flat.pdf

Life sciences study design

All studies must disclose on these points even when the disclosure is negative.

Sample size	Seven individuals, of 2 large families suffering of specific congenital eye malformation.
Data exclusions	Non excluded.
Replication	All data replicable.
Randomization	All available family members were included.
Blinding	Identifying disease associated locus and mutation required non blinded studies.

Reporting for specific materials, systems and methods

We require information from authors about some types of materials, experimental systems and methods used in many studies. Here, indicate whether each material, system or method listed is relevant to your study. If you are not sure if a list item applies to your research, read the appropriate section before selecting a response.

Materials & experimental systems

n/a	Involved in the study	
☒	☐	Antibodies
☐	☒	Eukaryotic cell lines
☒	☐	Palaeontology and archaeology
☐	☒	Animals and other organisms
☒	☐	Clinical data
☒	☐	Dual use research of concern

Methods

n/a	Involved in the study	
☒	☐	ChIP-seq
☒	☐	Flow cytometry
☒	☐	MRI-based neuroimaging

Eukaryotic cell lines

Policy information about cell lines and Sex and Gender in Research

Cell line source(s)	DT40 (Chicken bursal lymphoma cell line). The RPE used in this manuscript are primary cells, see later section.
Authentication	The DT40 cell line was obtained from ATCC.
Mycoplasma contamination	None: tested routinely.
Commonly misidentified lines (See ICLAC register)	None.

Animals and other research organisms

Policy information about studies involving animals; ARRIVE guidelines recommended for reporting animal research, and Sex and Gender in Research

Laboratory animals	Mice, C57BL/6N×C57BL/6N, E11.5 embryos. Chicken embryos (in ovo).
Wild animals	The study did not involved wild animals.
Reporting on sex	Sex of embryos is irrelevant and was not collected.
Field-collected samples	This study does not involved field collected samples.
Ethics oversight	Mouse enhancer assays were carried out in transgenic mouse embryos by Cyagen Biosciences (Cyagen US inc.), whose facility meets animal health and welfare guidelines. All chicken experimentation was performed in ovo. ATAC was performed on E4/E5 primary RPE, and electroporation was conducted in developing embryos. No IACUC / regulatory approval for chicken embryos was required, as they are not considered live animals by the relevant institution's regulatory bodies.

Note that full information on the approval of the study protocol must also be provided in the manuscript.
